# Systematic review of the relationship of *Helicobacter pylori* infection with geographical latitude, average annual temperature and average daily sunshine

**DOI:** 10.1186/s12876-018-0779-x

**Published:** 2018-04-17

**Authors:** Chao Lu, Ye Yu, Lan Li, Chaohui Yu, Ping Xu

**Affiliations:** 1 Department of Gastroenterology, the First Affiliated Hospital, College of Medicine, Zhejiang University, No. 79 Qingchun Road, Hangzhou, 310003 China; 2 Department of Rheumatology, the First Affiliated Hospital, College of Medicine, Zhejiang University, Hangzhou, 310003 China

**Keywords:** Latitude, Temperature, Sunshine, *Helicobacter pylori*

## Abstract

**Background:**

*Helicobacter pylori (H. pylori)* infection is a worldwide threat to human health with high prevalence. In this study, we analyzed the relationship between latitude, average annual temperature, average daily sunshine time and *H. pylori* infection.

**Methods:**

The PubMed, ClinicalTrials.gov, EBSCO and Web of Science databases were searched to identify studies reporting *H. pylori* infection. Latitude 30° was the cut-off level for low and mid-latitude areas. We obtained information for latitude, average annual temperature, average daily sunshine, and Human Development Index (HDI) from reports of studies of the relationships with *H. pylori infection*.

**Results:**

Of the 51 studies included, there was significant difference in *H. pylori* infection between the low- and mid-latitude areas (*P* = 0.05). There was no significant difference in the prevalence of *H. pylori* infection in each 15°-latitude zone analyzed (*P* = 0.061). Subgroup analysis revealed the highest and lowest *H. pylori* infection rates in the developing regions at > 30° latitude subgroup and the developed regions at < 30° latitude subgroup, respectively (*P* < 0.001). Multivariate analysis showed that average annual temperature, average daily sunshine time and HDI were significantly correlated with *H. pylori* infection (*P* = 0.009, *P* < 0.001, *P* < 0.001), while there was no correlation between *H. pylori* infection and latitude.

**Conclusions:**

Our analysis showed that higher average annual temperature was associated with lower *H. pylori* infection rates, while average daily sunshine time correlated positively with *H. pylori* infection. HDI was also found to be a significant factor, with higher HDI associated with lower infection rates. These findings provide evidence that can be used to devise strategies for the prevention and control of *H. pylori*.

**Electronic supplementary material:**

The online version of this article (10.1186/s12876-018-0779-x) contains supplementary material, which is available to authorized users.

## Background

*Helicobacter pylori* (*H. pylori*) is a Gram-negative microaerophilic bacterium that dwells in human gastric mucosa, causing stomach injury. *H. pylori* infection is commonly associated with gastroduodenal diseases in humans, such as chronic gastritis and peptic ulcers [1], gastric mucosa-associated lymphoid tissue lymphoma [2], and even gastric cancer [3, 4]. Almost 50% of the human population worldwide is infected, with a higher rate in people living in developing countries [5]. A large number of studies have provided evidence of *H. pylori* in dental plaques, houseflies, human and animal feces, and natural environmental waters [6]. Therefore, water supplies contaminated by sewage containing fluids or feces from infected people have been considered to be a potential route of *H. pylori* transmission [6]. Several factors may contribute to *H. pylori* infection, such as socioeconomic status and living conditions [7], metabolic syndrome [8], sex [9], education and smoking [10]. Among these factors, socioeconomic conditions play an important role in *H. pylori* infection. Our previous analysis of the Human Development Index (HDI) confirmed that high *H. pylori* recurrence rates are more likely in less-developed areas [7]. Thus, prevention and therapy of *H. pylori* have become a public health challenge.

There is clear geographic variation in the prevalence of *H. pylori* infection [11, 12]. Furthermore, vitamin D and vitamin D receptor (VDR) play an important protective role in *H. pylori* infection [13]. Vitamin D is an immunoregulatory agent widely known to mediate bone metabolism and plays a key role in target tissues, such as the kidney, thyroid, intestine, skin, immune cells, nonparenchymal hepatocytes, and biliary epithelial cells [14, 15]. Vitamin D synthesis depends on exposure to sunlight and solar ultraviolet radiation, which is affected by latitude, season, temperature and duration of daily sunshine [16]. Therefore, we hypothesized that the prevalence of *H. pylori* infection varies with changes in geographic areas (different latitudes, temperature and average daily sunshine time) that are associated with differences in the rates of vitamin D synthesis.

The purpose of this study was to determine the influence of different latitudes, temperature, HDI and average daily sunshine time on *H. pylori* infection rates. This information will highlight a novel epidemiologic and global perspective of *H. pylori* infection.

## Methods

### Search strategy and study selection

We searched articles published from January 1, 2000 to December 1, 2016 in the PubMed, ClinicalTrials.gov, EMBASE and Web of Science databases using the following search terms: (Helicobacter pylori OR H. pylori OR Helicobacter infection OR Helicobacter* OR HP OR Helicobacter pylori (MeSH))*, and* (*infection OR infectious (MeSH)*). Our study was limited to humans only and studies involving participants undergoing physical examination were included. People with digestive disease, such as gastritis, peptic ulcer, and stomach cancer, were excluded if they underwent physical examination. In addition, we focused on studies with participants aged over 18 years. Age, sex, smoking, HDI and other confounding factors were also considered. Eligibility was evaluated by two investigators independently. The quality of papers was assessed using the Strengthening the Reporting of Observational studies in Epidemiology (STROBE) checklist [17]. Any study-related disagreements were resolved by a third reviewer.

### Definitions

#### Diagnostic methods for *H. pylori* infection

Combining the guidelines [18] and previous meta-analysis [19], a diagnosis of *H. pylori* infection was confirmed on the basis of at least one positive result from the following tests: (1) ^13^C/^14^C urea breath test (UBT); (2) rapid urease test (RUT); (3) *H. pylori* culture; (4) stool antigen test; or (5) histology of biopsy staining. Because many medical centers used a serologic test for physical examination, serologic tests were also used to confirm diagnosis.

#### Average daily sunshine time, latitude, temperature and HDI

After identification of the geographical location of the city or area participating in the study, we used the Hong Kong Observatory (http://gb.weather.gov.hk/contentc.htm) to obtain information for calculation of the average daily sunshine time and the average daily temperature of every city included in this study. The average daily sunshine time was calculated according to the following equation:

Average daily sunshine time = annual average sunshine time/365; Annual average temperature was used in the analysis.

HDI was chosen to assess the socioeconomic status at the national level. This index is a measure of three basic dimensions of human development: health index (according to life expectancy at birth), education index (according to mean and expected years of schooling), and decent standard of living (gross national income per capita) [7]. The HDI data (1990–2012) is available on the United Nations Development Programme website (http://hdr.undp.org/en/reports/). Countries with a high HDI score (0.788 or higher) are regarded as developed, while others are defined as developing according to the United Nations [20].

### Data abstraction

Data were extracted to Microsoft Excel (2007 edition; Microsoft, Redmond, WA, USA) for effective organization. The following data were obtained from included studies: the study area, latitude, average annual temperature and average daily sunshine duration (according to study area or country), year of study, participant number, diagnostic method used for *H. pylori* infection, HDI levels (according to study country in the relevant years), age, sex, and smoking.

We excluded papers without information for latitude, average annual temperature or average daily sunshine time. All data were double-checked by two authors.

### Statistical analysis

The data obtained in this study exhibited normal distribution; therefore, Student’s *t*-test was used to compare numerical variables for each latitude zone and different HDI zones. One-way analysis of variance (ANOVA) was performed to compare multiple groups and analysis of covariance (ANCOVA) was performed to analyze influence effects. Pair-wise comparisons of multiple groups were performed with Bonferroni Correction [21] if necessary. In the multivariate analysis, stepwise linear regression analysis was used to correlate *H. pylori* infection with HDI, latitude, average annual temperature and average daily sunshine time. Adjustment and discretization of different variables were conducted if necessary. Moreover, the potential of sex, age and smoking as influencing factors was analyzed in a randomized controlled model (*I*^*2*^ > 50%) or a fixed controlled model (*I*^*2*^ ≤ 50%) to compare differences between infected and non-infected individuals. In the multivariate analysis, *P* ≤ 0.05 was considered to indicate a significant correlation and 0.1 > *P* > 0.05 was considered to indicate a suggestive correlation. For other statistical methods, *P* ≤ 0.05 was considered to indicate statistical significance. All statistical analysis was performed using SPSS 17.0 (IBM, Chicago, IL, USA). Associated data were calculated and plotted using GraphPad Prism 5 (Graph Pad, San Diego, CA, USA). The randomized controlled model was performed using Stata 12.0 (StataCorp, College Station, TX, USA).

## Results

### Study selection

**Fig. 1:**
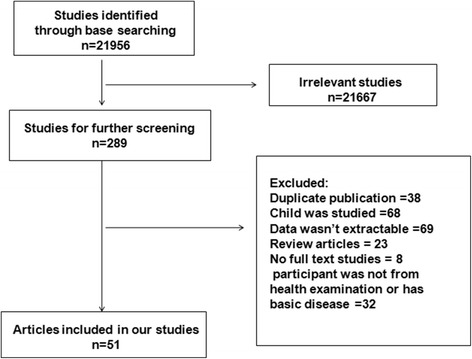
Flow diagram of search strategy and study criteria

Fifty-one studies originating from 58 regions were included in our final analysis (Fig. 1). Primary data and results for author, study area, HDI, latitude, average annual temperature, and average daily sunshine time are listed in Table 1. Geographically, studies originated mainly from Asia (25/58), Europe (19/58), America (11/58), Africa (2/58) and Oceania (1/58). In addition, according to the dichotomy of HDI, additional studies were included from developed countries (32/58) and developing countries (26/58). The cut-off level between the low and mid-latitude was set at 30° latitude. Based on latitude, 13 studies originated from low latitude zones, and 45 from zones 30° latitude and higher. Statistically significant heterogeneity was observed among all studies in this analysis.

**Table 1:** The basic characteristics of included papers

Region	Author	Number (n)	Positive rate	Latitude (°)	Temperature (°C)	Sunshine (h)	HDI
Europe							
Israel	Niv	2128	0.328	31.00	9.31	16.45	0.872
Nottingham	Jackson	2437	0.264	52.56	3.77	9.80	0.768
Lebanon	Naja	308	0.520	33.45	8.05	20.87	0.761
Berlin	Berg	1806	0.392	52.30	4.45	8.88	0.801
Rome	Gasbarrini	655	0.400	41.80	6.77	15.20	0.825
Leeds	Moayyedi	8429	0.276	53.48	3.36	8.76	0.818
Magdeburg	Wex	2318	0.444	52.80	4.45	8.75	0.906
Novosibirsk	Reshetnikov	438	0.884	55.20	6.03	1.73	0.723
Prague	Bures	1406	0.292	50.05	4.57	7.85	0.867
Loiano	Bazzoli	1533	0.679	44.16	5.59	8.60	0.803
Stockholm	Sorberg	3502	0.177	59.19	4.99	6.63	0.855
Wroclaw	Iwanczak	3307	0.842	51.10	4.10	8.32	0.794
Tbilisi	Kretsinger	125	0.719	41.70	5.63	13.00	0.690
Heidelberg	Michel	1797	0.481	49.25	4.49	11.50	0.916
Bratislava	Kuzela	1838	0.351	48.08	5.58	10.50	0.836
Tirana	Monno	1088	0.707	41.19	6.97	15.20	0.682
Reykjavik	Thjodleifsson	447	0.363	64.08	3.48	4.30	0.859
Uppsala	Thjodleifsson	359	0.112	59.51	4.86	6.50	0.897
Tartu	Thjodleifsson	240	0.692	58.23	4.59	4.84	0.780
Asia							
Beijing	Zhang	2006	0.833	40.15	7.52	11.80	0.645
Ankara	Akin	1089	0.774	39.52	6.71	11.71	0.653
Korea	Yim	13,697	0.586	36.00	5.77	11.82	0.853
Okinawa	Toyoda	1540	0.599	26.50	5.15	22.42	0.871
Malaysia	Goh	2381	0.359	4.00	6.11	26.73	0.727
Islamabad	Rasheed	205	0.819	33.43	8.07	21.34	0.522
Yangzhong	Zhu	5417	0.634	32.19	5.85	15.10	0.699
North Sulawesi	Miftahussurur	251	0.143	1.29	6.00	27.72	0.684
Arak	Afsharipour	525	0.742	34.10	8.09	13.65	0.751
Nahavand	Alizadeh	1518	0.710	34.11	7.55	10.83	0.735
Penang	Sasidharan	5370	0.142	5.24	6.75	27.00	0.723
Tehran	Nouraie	2326	0.690	35.40	8.25	17.00	0.703
Seoul	Kim	1485	0.649	37.33	5.77	11.82	0.853
Kota Bharu	Rahim	480	0.190	6.90	6.94	26.73	0.769
Hsinchu	Chen	3578	0.202	24.81	5.07	22.60	0.882
Xiangshui	Shi	1371	0.620	34.20	6.57	15.79	0.641
Korea	Lim	10,796	0.545	36.00	5.77	11.82	0.891
Beijing	Cheng	1232	0.468	40.15	7.52	11.80	0.812
Hangzhou	Xu	8820	0.438	30.30	5.42	15.79	0.723
Hokkaido	Ueda	1428	0.294	43.14	4.94	8.22	0.890
Aomori	Ueda	782	0.497	40.49	4.64	9.73	0.890
Yamagata	Ueda	3615	0.545	38.30	4.56	11.19	0.890
Gunma	Ueda	4914	0.323	36.40	5.42	13.91	0.890
Aichi	Ueda	2237	0.306	35.10	5.58	15.04	0.890
Kagawa	Ueda	442	0.378	34.30	5.80	15.35	0.890
America							
America	Everhart	7465	0.325	36.09	7.07	15.06	0.859
Nashville	Epplein	310	0.787	36.09	6.88	15.06	0.888
Seattle	Ioannou	6724	0.535	47.38	5.95	11.13	0.859
Ontario	Naja	1306	0.294	43.40	5.58	7.16	0.896
Aklavik	Cheung	194	0.660	68.13	3.54	−8.20	0.896
Nassau	Carter	204	0.578	24.15	7.91	24.85	0.778
São Paulo	Zaterka	993	0.657	23.33	5.49	19.20	0.720
Guadeloupe	Weill	854	0.552	16.15	7.60	26.30	0.848
São Paulo	Oba–Shinjo	942	0.484	23.33	4.75	19.26	0.688
Recife	Melo	405	0.314	8.30	6.75	25.46	0.683
Pelotas	Santos	359	0.644	31.46	6.14	17.50	0.709
Africa							
Belgium	Aguemon	446	0.740	6.21	6.44	27.22	0.413
Tunis	Mansour	250	0.632	36.48	7.69	20.00	0.689
Oceania							
Queensland	Pandeya	1316	0.230	27.30	7.50	21.40	0.904

*HDI* Human Development Index

### Prevalence of *H. pylori* infection

**Fig. 2:**
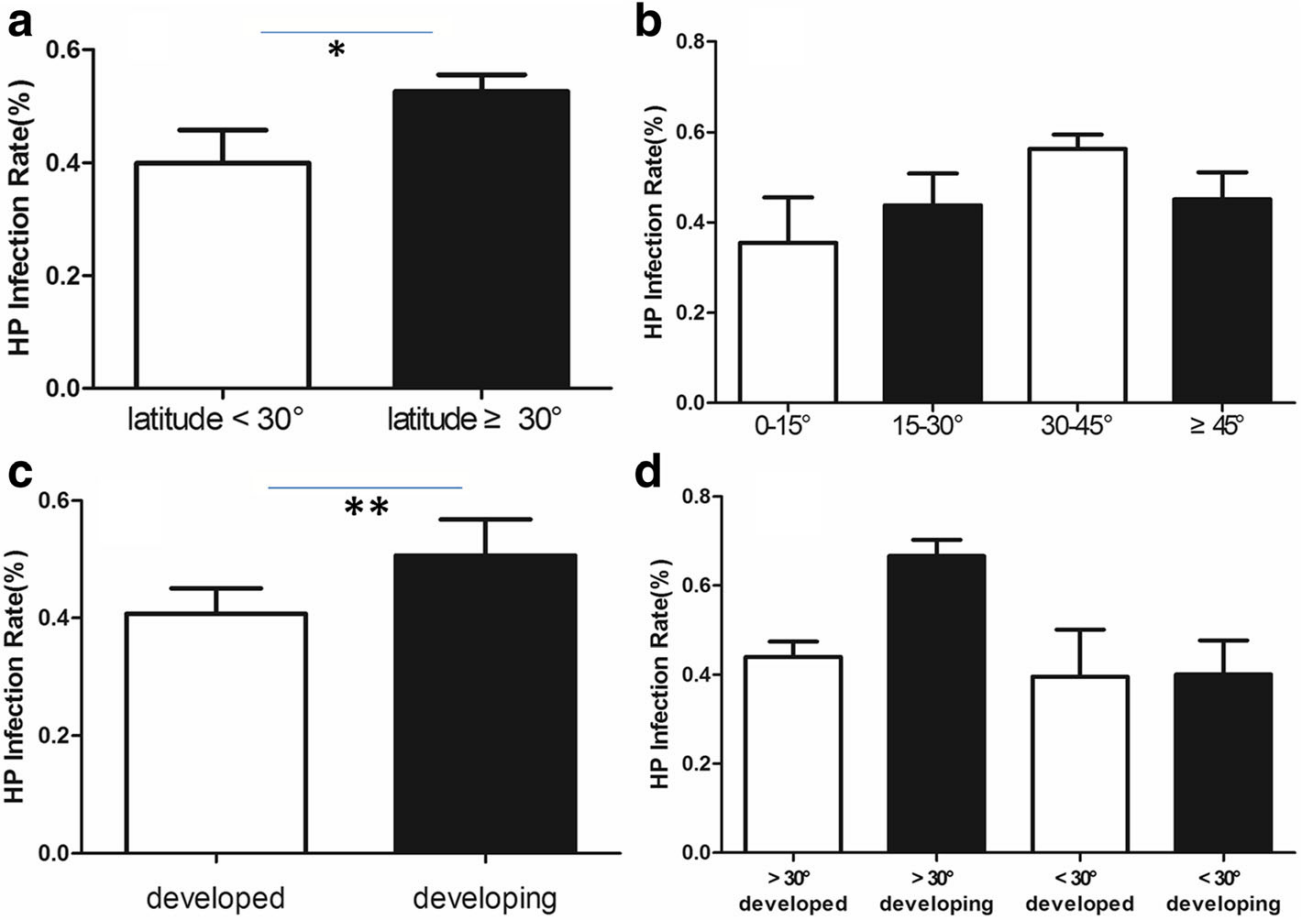
**a** Comparison of the prevalence of *Helicobacter pylori* infection between low and mid-to-high latitude zones (39.92% ± 21.15% vs. 52.56% ± 19.88%, *P = 0.05); **b** Comparisons of the prevalence of *H. pylori* infection in each 15°-latitude zone; **c** Comparison of the prevalence of *H. pylori* infection between developed and developing regions (43.48% ± 17.73% vs. 57.42% ± 21.76%, **P = 0.009); **d** Comparisons of the prevalence of *H. pylori* infection in developed countries and mid-to-high latitude zones, developing countries and mid-to-high latitude zones, developed countries and low latitude, developing countries and low latitude zones (*P* < 0.001)

**Fig. 3:**
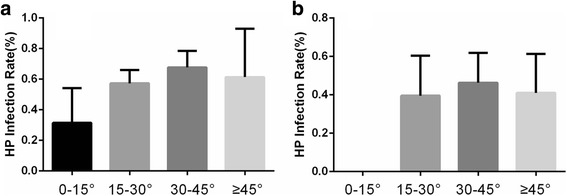
**a** Comparisons of the prevalence of *Helicobacter pylori* infection for every 15°-latitude zone in developing regions; **b** Comparisons of the prevalence of *H. pylori* infection for every 15°-latitude zone in developed regions

The overall prevalence of *H. pylori* infection was 49.73% ± 20.68%, with a significant difference in the prevalence of *H. pylori* infection between the latitude zones < 30° and ≥ 30° (39.92% ± 21.15% vs. 52.56% ± 19.88%, *P* = 0.05) (Fig. 2a). We further analyzed the prevalence of *H. pylori* infection in every 15°-latitude zone. The *H. pylori* infection rate was 35.43% ± 24.68% (0–15°), 43.77% ± 18.71% (15–30°), 56.29% ± 17.24% (30–45°), and 45.10% ± 23.18% (≥ 45°), respectively. ANOVA did not show a significant difference in the prevalence of *H. pylori* infection for each latitude zone (*P* = 0.061). However, we observed a rising trend in the prevalence of *H. pylori* infection from latitude 0° to 45° (Fig. 2b). Most of the regions in the latitude ≥45° zones are developed areas; therefore, we used ANCOVA to assess the existence of confounding effects caused by the HDI. The results showed a linear relationship between HDI and latitude (F = 22.328, *P* < 0.001) and HDI affected the result of latitude as a confounding factor (*P* = 0.001). We then conducted a subgroup analysis. In the developing and developed regions, we also observed a similar rising trend in the prevalence of *H. pylori* infection from latitude 0 to 45° (Fig. 3a and b). We also divided regions into developed and developing regions according to the HDI. Individuals living in developed regions showed a lower infection rate than those in the developing regions (43.48% ± 17.73% vs. 57.42 ± 21.76%, *P* = 0.009) (Fig. 2c). To further confirm the relationship between HDI, latitude and *H. pylori* infection, we determined *H. pylori* infection rates in the following four groups of areas: I): 44.03% ± 17.60% (developed countries & > 30° latitude); II): 66.60% ± 15.08% (developing countries & > 30° latitude); III): 39.58% ± 20.88% (developed countries & < 30° latitude); and IV): 40.08% ± 22.53% (developing countries & < 30° latitude) (Fig. 2d); there were significant differences between each group (*P* < 0.001). The *H. pylori* infection rate was highest in developing regions at > 30° latitude, while the rate was lowest in developed regions at < 30° latitude. This indicated that low latitude regions with high HDI is protective against *H. pylori* infection.

Stepwise linear regression analysis revealed that HDI was significantly correlated with the prevalence of *H. pylori* infection (coefficient = − 0.556, *P* < 0.001). Furthermore, higher average annual temperature correlated with lower infection rates (coefficient = − 0.577, *P* < 0.001) and higher average daily sunshine time correlated with higher *H. pylori* infection rates (coefficient = 0.342, *P* = 0.009). However, there was no significant correlation between the prevalence of *H. pylori* infection and latitude. In European regions, we found that only HDI affected the prevalence of *H. pylori* infection (coefficient = − 0.648, *P* = 0.003), while the prevalence in Asian regions was affected by both HDI (coefficient = − 0.584, *P* < 0.001) and latitude (coefficient = 0.744, *P* < 0.001). Moreover, through adjustment and discretization of HDI, we also observed that latitude (coefficient = 0.774, *P* < 0.001) exerts an independent effect on the prevalence of *H. pylori* infection.

Based on existing data, we conducted meta-analysis to analyze the influences of age, sex, smoking and education on the *H. pylori* infection rate. *H. pylori*-infected individuals were found to be significantly older than non-infected infected individuals (SMD = 0.26, 95% CI = 0.09–0.44, *I*^*2*^ = 94.2%, *P* < 0.01; Additional file 1) and males were 1.1 times more likely to be infected with *H. pylori* than females (OR = 1.1, 95% CI = 1.04–1.16, *I*^*2*^ = 72.1%, *P* < 0.01; Additional file 1). Furthermore, individuals with a high level of education were 0.58 times more likely to be infected with *H. pylori* than individuals with a low level of education (OR = 0.58, 95% CI = 0.41–0.84, *I*^*2*^ = 97.1%, *P* < 0.01; Additional file 1). In contrast, there were no significant differences in the *H. pylori infection rates* between smokers and non-smokers (OR = 0.99, 95% CI = 0.93–1.05, *I*^*2*^ = 43.3%, *P* = 0.042; Additional file 1).

## Discussion

To the best of our knowledge, this is the first study investigating the relationships between *H. pylori* infection and latitude, average annual temperature and average daily sunshine time. Our multiple-factor analysis showed that higher average annual temperature was associated with lower *H. pylori* infection rates, while average daily sunshine time correlated positively with *H. pylori* infection. HDI was also found to be a significant factor, with higher HDI associated with lower infection rates.

We found higher *H. pylori* infection rates among the populations in high latitude regions. Furthermore, we observed a trend of increasing infection rates with increasing latitude. Vitamin D is known to be closely linked to ultraviolet radiation exposure [22] and lower latitudes are characterized by stronger ultraviolet radiation. Therefore, people living in low latitude zones may generate higher levels of vitamin D, which played a protective role in *H. pylori* infection, as shown by us previously [13]. In addition, Kwon et al. reported that vitamin D induced expression of vitamin D3-upregulated protein 1 (VDUP1), which reduced *H. pylori*-induced gastric carcinogenesis in mice [23]. The vitamin D nuclear receptor, which binds vitamin D, is detected in gastric mucosa [24]. Furthermore, the function of vitamin D in antimicrobial innate immune responses [25] indicates the possibility of a role in reducing *H. pylori* infection. In general, it can be speculated that different latitudes affect the synthesis of vitamin D, with a consequential influence on *H. pylori* infection rates. Our study also showed a suggestive correlation between latitude and infection rate, although no significant correlation was identified in our multiple-factor analysis. Interestingly, however, we observed that latitude independently affected *H. pylori* infection in Asian countries, indicating that latitude is an extremely important factor in *H. pylori* infection in these regions. Therefore, further studies are required to support the hypothesis that latitude influences *H. pylori* infection rates in different geographical regions.

In this study, we also found that average annual temperature was significantly related to *H. pylori* infection, a link that has not been reported previously. We propose that suitably warm temperatures provide more opportunities for people to engage in outside activities, with increased vitamin D synthesis resulting from the increased exposure to sunlight. However, this hypothesis requires verification in large-scale epidemiological studies and basic research.

Our results showed that more average daily sunshine time is associated with higher *H. pylori* infection rate, which was contrary to our expectation based on the positive correlation of daily sunshine time with vitamin D synthesis. Daily sunshine time is determined by several factors such as altitude, active area, environment. Our findings may provide evidence that ultraviolet light intensity based on latitude has a much more important influence on *H. pylori* infection than ultraviolet light exposure time. Furthermore, the *H. pylori* infection rate may also be influenced by other factors, such as sex, age, and education.

HDI, which represents the gold standard for measurement of human development, was found to be inversely related to *H. pylori* infection, which is consistent with a previous epidemiological study [26]. In our study, we found lower *H. pylori* infection rates in regions with latitude > 45°, which was not consistent with the trend of increasing *H. pylori* infection rates with latitude. Globally, regions higher than 45° latitude contain mainly the developed countries of North America and Northern Europe; thus, indicating that HDI plays a predominant role in the lower *H. pylori* infection rates found in these regions. ANCOVA showed a linear relationship between HDI and latitude and that HDI had a distinct influence on the effect of latitude on *H. pylori* infection rates. Furthermore, we observed the lowest infection rate among individuals living in developed regions with low latitude, which further illustrated that the importance of latitude and HDI on the rate of *H. pylori* infection.

Due to the lack of related reports, the results of our analysis of the influence of smoking were inconsistent with those of previous studies [27]; further investigations are required for clarification of the influence of this factor on the rate of *H. pylori* infection.

To our knowledge, this is the first study of the association of *H. pylori* infection with geographical latitude, average daily sunshine time, and average annual temperature.

Despite the strength of the numbers of participants, some limitations of our study should be noted. First, the studies included in our analysis predominantly used two diagnostic methods: the ^13^C UBT and the serologic test; however, different diagnostic methods have different positive diagnostic rates [28]. The specificity and sensitivity of serologic tests were 100% and 82% respectively, while the corresponding values for the ^13^C UBT were 100% and 92%, respectively [29]. Therefore, the differences in diagnostic approach may lead directly to selection bias in the included participants and consequently, to a greatly increased false positive rate. The ^13^C UBT is the most effective noninvasive diagnostic method for detection of *H. pylori* infection with high specificity and sensitivity that is currently available. Second, our definition of latitude, average daily sunshine time and average annual temperature were based on the areas in which the research originated, while it was not certain that the participants really represented the area selected. Selected participants originating from different areas introduce a selection bias. In addition, although all the participants were healthy and without any digestive diseases during the study, it was not clear whether previous disease or other systemic diseases may have influenced *H. pylori* infection. Finally, several original studies failed to adjust for potentially confounding factors such as race, environmental factor, and gene polymorphisms. Any of these factors can lead to bias in the results.

## Conclusions

In conclusion, we have demonstrated that *H. pylori* infection is significantly related to average annual temperature, average daily sunshine time and HDI. Higher average annual temperature and HDI correlated with lower *H. pylori* infection rates. Average daily sunshine time correlated positively with *H. pylori* infection rates; however, no correlation between the prevalence of *H. pylori* infection and latitude was observed in the multivariate analysis. Nevertheless, individuals living in high latitude regions showed a high infection rate. In consideration of the influence of HDI, a suggestive increasing trend of *H. pylori* infection rate with rising latitude also existed. Moreover, the combined statistically significant differences in infection rates at different latitudes and HDI scores suggest that latitude is also an influencing factor. Therefore, populations living in regions with low average annual temperature, low HDI and high latitude need to be alerted to the risk of *H. pylori* infection. We also believe that the global prevalence of *H. pylori* infection should be evaluated from a human development perspective. Our findings require further verification in large-scale epidemiological investigations.

## Additional file

Additional file 1: Meta-analysis of analyzing the influences of age, sex, smoking and education on the *H. pylori* infection rate. *H. pylori*-infected individuals were older than non-infected infected individuals (SMD = 0.26, 95% CI = 0.09–0.44, I2 = 94.2%, *P* < 0.01) and males were 1.1 times more likely to be infected with *H. pylori* than females (OR = 1.1, 95% CI = 1.04–1.16, I2 = 72.1%, *P* < 0.01). High-educated individuals were 0.58 times more likely to be infected with *H. pylori* than low-educated individuals (OR = 0.58, 95% CI = 0.41–0.84, I2 = 97.1%, *P* < 0.01). There were no significant differences in the *H. pylori* infection rates between smokers and non-smokers (OR = 0.99, 95% CI = 0.93–1.05, I2 = 43.3%, *P* = 0.042). (PDF 155 kb)

## Abbreviations

*H. pylori*: *Helicobacter pylori*
HDI: Human development index
RUT: Rapid urease test
UBT: Urea breath test
VDR: Vitamin D receptor
VDUP1: Vitamin D3-upregulated protein 1
