# Supplementary material for: Systematic review of the relationship of Helicobacter pylori infection with geographical latitude, average annual temperature and average daily sunshine
Source: BMC Gastroenterol. 2018 Apr 17;18:50. doi: 10.1186/s12876-018-0779-x (PMC5905136; doi:10.1186/s12876-018-0779-x)
Supplement: Supplementary file 1 — Meta-analysis of analyzing the influences of age, sex, smoking and education on the H. pylori infection rate. H. pylori-infected individuals were older than non-infected infected individuals (SMD = 0.26, 95% CI = 0.09–0.44, I2 = 94.2%, P < 0.01) and males were 1.1 times more likely to be infected with H. pylori than females (OR = 1.1, 95% CI = 1.04–1.16, I2 = 72.1%, P < 0.01). High-educated individuals were 0.58 times more likely to be infected with H. pylori than low-educated individuals (OR = 0.58, 95% CI = 0.41–0.84, I2 = 97.1%, P < 0.01). There were no significant differences in the H. pylori infection rates between smokers and non-smokers (OR = 0.99, 95% CI = 0.93–1.05, I2 = 43.3%, P = 0.042). (PDF 155 kb) [file 12876_2018_779_MOESM1_ESM.pdf]

Meta-analysis of analyzing the influences of age, sex, smoking and education on the *H. pylori* infection rate.

Age

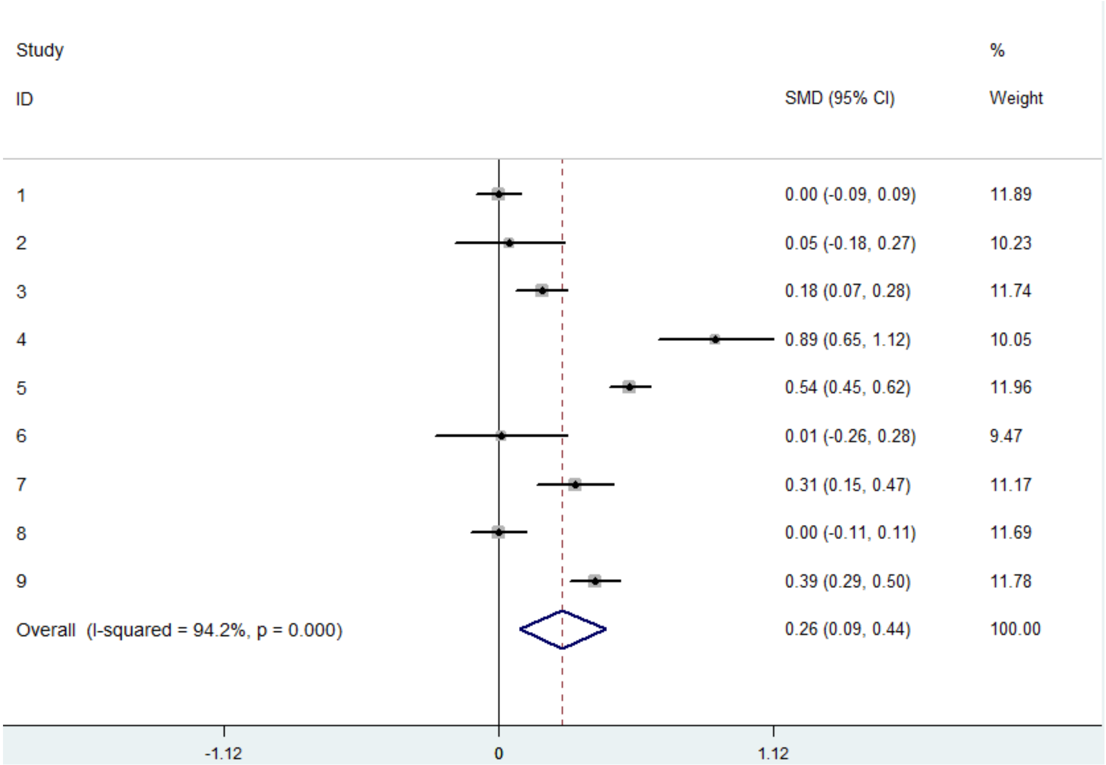

Education

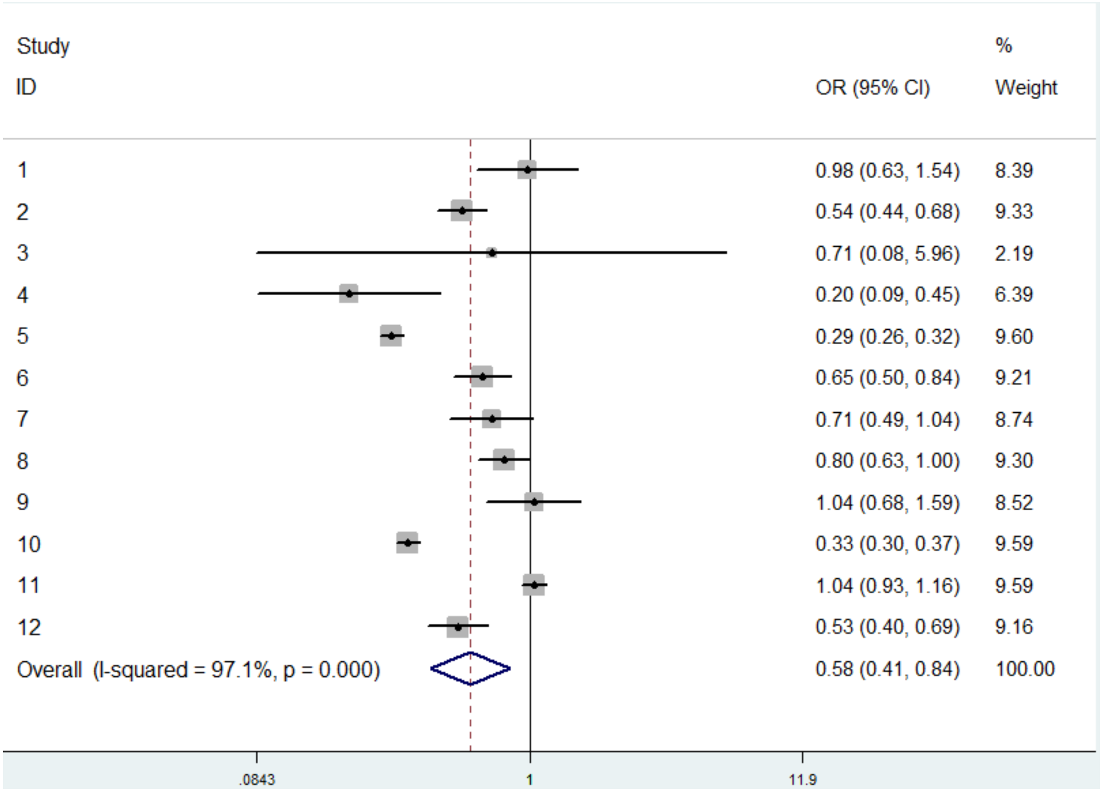

Smoking

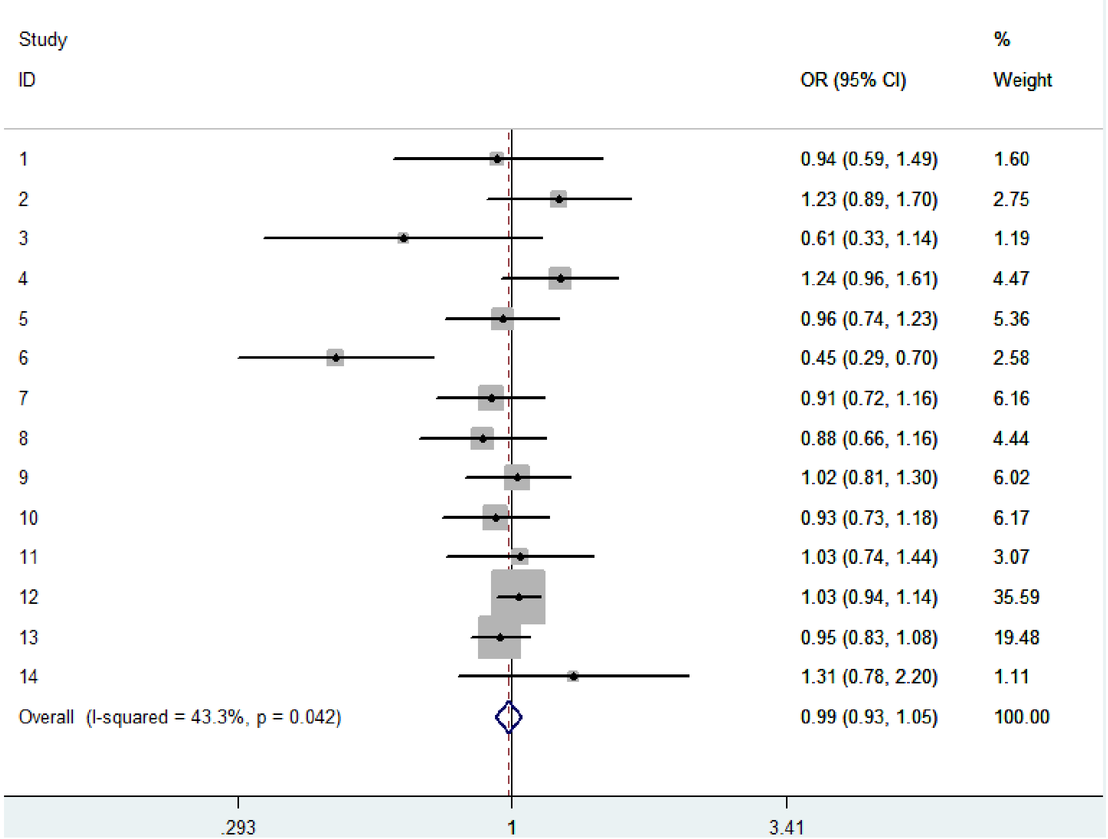
Study
ID
OR (95% CI)
%
Weight
1 0.94 (0.59, 1.49) 1.60
2 1.23 (0.89, 1.70) 2.75
3 0.61 (0.33, 1.14) 1.19
4 1.24 (0.96, 1.61) 4.47
5 0.96 (0.74, 1.23) 5.36
6 0.45 (0.29, 0.70) 2.58
7 0.91 (0.72, 1.16) 6.16
8 0.88 (0.66, 1.16) 4.44
9 1.02 (0.81, 1.30) 6.02
10 0.93 (0.73, 1.18) 6.17
11 1.03 (0.74, 1.44) 3.07
12 1.03 (0.94, 1.14) 35.59
13 0.95 (0.83, 1.08) 19.48
14 1.31 (0.78, 2.20) 1.11
Overall (I-squared = 43.3%, p = 0.042) 0.99 (0.93, 1.05) 100.00
.293
1
3.41

Gender

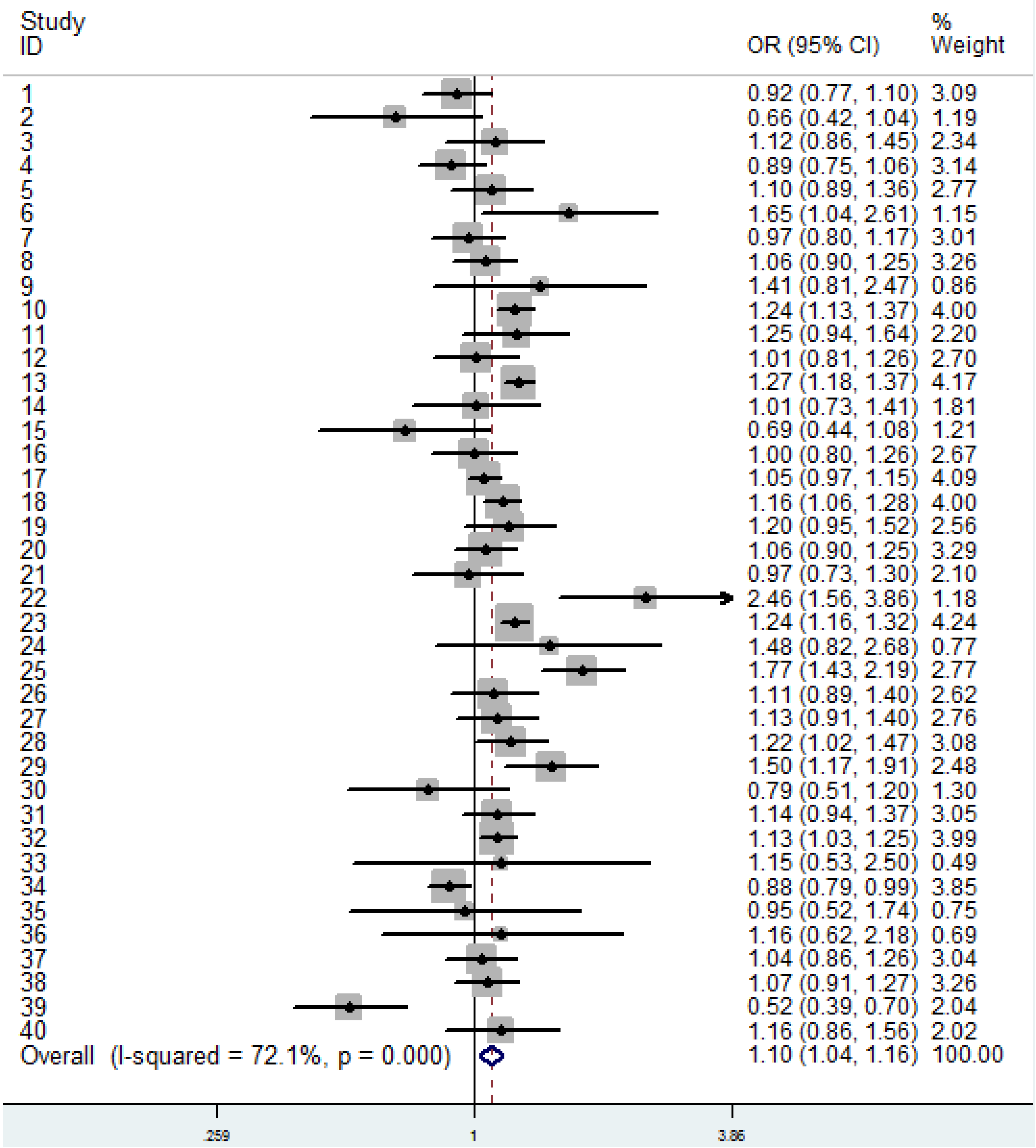
Study ID
OR (95% CI)
% Weight
1 0.92 (0.77, 1.10) 3.09
2 0.66 (0.42, 1.04) 1.19
3 1.12 (0.86, 1.45) 2.34
4 0.89 (0.75, 1.06) 3.14
5 1.10 (0.89, 1.36) 2.77
6 1.65 (1.04, 2.61) 1.15
7 0.97 (0.80, 1.17) 3.01
8 1.06 (0.90, 1.25) 3.26
9 1.41 (0.81, 2.47) 0.86
10 1.24 (1.13, 1.37) 4.00
11 1.25 (0.94, 1.64) 2.20
12 1.01 (0.81, 1.26) 2.70
13 1.27 (1.18, 1.37) 4.17
14 1.01 (0.73, 1.41) 1.81
15 0.69 (0.44, 1.08) 1.21
16 1.00 (0.80, 1.26) 2.67
17 1.05 (0.97, 1.15) 4.09
18 1.16 (1.06, 1.28) 4.00
19 1.20 (0.95, 1.52) 2.56
20 1.06 (0.90, 1.25) 3.29
21 0.97 (0.73, 1.30) 2.10
22 2.46 (1.56, 3.86) 1.18
23 1.24 (1.16, 1.32) 4.24
24 1.48 (0.82, 2.68) 0.77
25 1.77 (1.43, 2.19) 2.77
26 1.11 (0.89, 1.40) 2.62
27 1.13 (0.91, 1.40) 2.76
28 1.22 (1.02, 1.47) 3.08
29 1.50 (1.17, 1.91) 2.48
30 0.79 (0.51, 1.20) 1.30
31 1.14 (0.94, 1.37) 3.05
32 1.13 (1.03, 1.25) 3.99
33 1.15 (0.53, 2.50) 0.49
34 0.88 (0.79, 0.99) 3.85
35 0.95 (0.52, 1.74) 0.75
36 1.16 (0.62, 2.18) 0.69
37 1.04 (0.86, 1.26) 3.04
38 1.07 (0.91, 1.27) 3.26
39 0.52 (0.39, 0.70) 2.04
40 1.16 (0.86, 1.56) 2.02
Overall (I-squared = 72.1%, p = 0.000) 1.10 (1.04, 1.16) 100.00
.259
1
3.86
